# Zinc Deficiency–Associated Dermatitis in Infants During a Nationwide Shortage of Injectable Zinc — Washington, DC, and Houston, Texas, 2012–2013

**Published:** 2014-01-17

**Authors:** Duke Ruktanonchai, Michael Lowe, Scott A. Norton, Tiana Garrett, Lamia Soghier, Edward Weiss, June Hatfield, Jeffrey Lapinski, Steven Abrams, Wanda Barfield

**Affiliations:** 1 EIS officers, CDC; 2 Dept of Dermatology, Children’s National Medical Center, Washington, DC; 3 Dept of Neonatology, Children’s National Medical Center, Washington, DC; 4 Scientific Education and Professional Development Office, Office of Public Health Scientific Svcs, CDC; 5 Texas Children’s Hospital, Houston, Texas; 6 Div of Reproductive Health, National Center for Chronic Disease Prevention and Health Promotion. CDC

Injectable zinc, a vital component of parenteral nutrition (PN) formulations, has been in short supply in the United States since late 2012. In December 2012, three premature infants with cholestasis hospitalized in Washington, DC, experienced erosive dermatitis in the diaper area and blisters on their extremities, a condition that can be associated with zinc deficiency (1). All three infants were receiving PN because they had extreme cholestasis and were unable to be fed by mouth or tube. The PN administered to each infant was zinc deficient. Injectable zinc normally is added to PN for premature or medically compromised infants (e.g., those with cholestasis) by the hospital pharmacy because the amount of zinc needed by each patient differs; however, the pharmacy had run out of injectable zinc. No alternatives were available; other preparations of parenteral trace elements either contained insufficient zinc to meet infants’ requirements or had the potential to cause trace element toxicity in infants with cholestasis (2). The dermatitis of one infant resolved after the patient was able to take nutrition by mouth. The other two infants were found to have low serum zinc levels. In January 2013, CDC was notified of four additional cases of zinc deficiency among infants with cholestasis who received zinc-deficient PN in a hospital in Houston, Texas. In collaboration with the Food and Drug Administration (FDA), the two hospitals obtained emergency shipments of injectable zinc. No additional cases were reported. Current injectable zinc supplies have been increasing as FDA collaborates with pharmaceutical companies to import emergency supplies. FDA is working to establish temporary backup sources should future shortages occur.

On December 18, 2012, three cases of zinc deficiency disorder in premature infants were diagnosed in Washington, DC. Among the three infants, two were born at 24 weeks’ gestation, and one was born at 29 weeks’ gestation. Birth weights ranged from 551 g to 734 g (Table). The hospital caring for the infants had exhausted its supply of injectable zinc in November 2012. Infants typically receive injectable zinc and other trace elements as part of PN. Because of extreme cholestasis and prematurity in all three infants, they were unable to receive zinc through oral or enteral feedings. Among cholestatic infants, other preparations of zinc-containing parenteral trace elements might cause trace element toxicity; therefore, no alternatives to the injectable zinc supplements were available. On January 3, 2013, the District of Columbia Department of Health and CDC began case investigations. For this investigation, a case of zinc deficiency disorder was defined as an infant receiving zinc-deficient PN who had either a below-normal serum zinc level (<70 *μ*g/dL) or dermatitis consistent with zinc deficiency disorder. The objectives of the investigation were to 1) identify and describe cases of zinc deficiency disorder among infants at greatest risk for zinc deficiency, including those who were born at <37 weeks’ gestation, those weighing <1,500 g at birth, and those with chronic or permanent gastrointestinal dysfunction; 2) investigate the cause of the injectable zinc shortage; and 3) describe the need to monitor symptoms of micronutrient deficiency during micronutrient shortages.

After consulting with CDC, on January 10, 2013, the American Academy of Pediatrics (AAP) informed its members, which include approximately 3,000 neonatologists and 800 neonatal intensive care units nationwide, of the injectable zinc shortage. The members were asked to report to AAP and their respective state health department if their hospitals were experiencing a critical shortage of zinc. AAP compiled and forwarded all responses to CDC. On January 21, a neonatologist in Houston, Texas, reported four additional cases of zinc deficiency disorder to CDC. Among these four infants, two were born at gestational ages >37 weeks; one was born at 33 weeks, and one was born at 25 weeks. All four infants had cholestasis. Birth weights of these four infants ranged from 690 g to 2,950 g (Table). The Houston hospital also had exhausted its supplies of injectable zinc in November 2012. By January 22, 2013, a total of 17 hospitals in 10 states had reported shortages of zinc and other micronutrients. No additional cases of zinc deficiency disorder were identified. By the end of January, FDA was able to facilitate emergency shipment of injectable zinc to all 17 hospitals.

Each of the seven infants experienced zinc deficiency disorder after receiving zinc-deficient PN as a result of the nationwide shortage. The time from initiation of PN to diagnosis of zinc deficiency disorder ranged from 4 to 34 weeks; the exact number of weeks each infant was on zinc-deficient PN is unknown. Six patients were characterized as having low serum zinc levels (range:14–56 *μ*g/dL [normal: 70–120 *μ*g/dL]) and low alkaline phosphatase levels (range: 32–125 U/L [normal: 150–420 U/L]). Alkaline phosphatase levels typically are high in patients with cholestasis, but the zinc deficiency disorder in these infants resulted in low alkaline phosphatase levels. The serum zinc level for the first patient was not measured because suspicion of zinc deficiency disorder occurred after the infant had taken oral feedings containing zinc and the dermatitis had resolved.

All seven infants had cholestasis. Six of the seven had dermatitis consistent with zinc deficiency disorder, and three experienced bacterial infections. One infant experienced recurrent sepsis and liver failure before receiving zinc-deficient PN. This infant did not have dermatitis but had a low serum zinc level (Table). The infant died, and an autopsy was not performed. It is uncertain whether zinc deficiency disorder had a role in his death. After an emergency shipment of zinc was received by two hospitals, the remaining six infants received zinc in their PN, and all six infants improved clinically. Zinc and alkaline phosphatase levels returned to normal ranges, and the infants’ skin lesions resolved. Five of the six infants were discharged home. One infant remained hospitalized for 6 months for treatment of conditions unrelated to zinc deficiency disorder; that infant died in October 2013 from conditions unrelated to zinc deficiency disorder.

According to FDA, only two domestic manufacturers’ injectable zinc compounds are used in PN (American Regent and Hospira, Inc.). In fall 2012, American Regent informed FDA that it would be experiencing shortages of multiple chemicals, including zinc sulfate, because of delays in manufacturing. These delays resulted from drug quality concerns identified by the company, which included problems of particulate matter in the injectable products. FDA then contacted Hospira to determine whether they could meet the increase in zinc demand. Hospira representatives stated that the company was operating at maximum capacity and was unable to meet the increased demand; thus, the shortage continued to worsen in early January 2013. American Regent was able to release injectable zinc sulfate on January 22, but shortages continued. Hospira planned to release injectable zinc sulfate again by the end of 2013. One foreign manufacturer, Laboratoire Aguettant, and its authorized U.S. distributor, Baxter Healthcare, in conjunction with the FDA, have initiated temporary importation of an injectable zinc solution into the U.S. market to address this shortage.

FDA is continuing to work with American Regent and Hospira to expedite release of injectable zinc sulfate. Information for health-care providers regarding the zinc shortages and expected release dates of new supplies of injectable zinc is provided by FDA on its drug shortage website (http://www.fda.gov/drugs/drugsafety/drugshortages/ucm050792.htm). Finally, FDA is working with pharmaceutical companies to establish temporary backup sources for micronutrients should future shortages occur. When FDA uses regulatory discretion to allow a company to import a drug temporarily during a shortage, FDA ensures that the overseas manufacturing facility meets FDA quality standards and that its products (in terms of formulation and labeling) do not present undue risks for patients.

## Editorial Note

Zinc is an essential trace element that functions as a cofactor for certain enzymes involved in metabolism and cell growth (2,3); zinc supports immune function, protein metabolism, development of the gastrointestinal tract, and genetic processes (3). Acute zinc deficiency disorder is characterized by dermatitis around the limbs and body orifices, diarrhea, and impaired immune function, whereas chronic zinc deficiency disorder can lead to liver or kidney failure (2). A rare genetic disorder, acrodermatitis enteropathica, shares the same clinical manifestations as acute zinc deficiency disorder but is a metabolic disorder of zinc absorption. Zinc is a standard component in PN. Premature infants administered PN require 400 *μ*g/kg/body weight/day of zinc to maintain serum levels and promote growth, whereas 200 *μ*g/kg/body weight/day of zinc is sufficient for full-term infants on PN (4). Additionally, PN might be needed for prolonged periods for very low birth weight infants (<1,500 g) and infants with chronic gastrointestinal dysfunction.

What is already known on this topic?

Nationwide shortages of parenteral micronutrients have continued to occur in recent years. These shortages can lead to clinically significant micronutrient deficiencies among patients who depend on prolonged parenteral nutrition. Premature infants are especially vulnerable, and certain micronutrient deficiencies can be lethal.

What is added by this report?

The nationwide shortage of injectable zinc that began in late 2012 led to seven reported cases of zinc deficiency disorder in vulnerable infants. Among these infants, six experienced severe dermatitis, and three experienced invasive bacterial infections. The Food and Drug Administration is now temporarily permitting the importation and sale of an injectable zinc product.

What are the implications for public health practice?

Hospitals with limited stocks of injectable zinc should consider reserving supplies for infants with the highest risk for deficiency (e.g., those who are premature [born at <37 weeks’ gestation] or have very low birth weight [<1,500 g] and those with chronic or permanent gastrointestinal dysfunction). If shortages occur, monitoring patients on parenteral nutrition for signs and symptoms of micronutrient deficiencies is crucial.

Studies have reported progressively decreasing serum zinc levels among infants on zinc-deficient PN, particularly premature infants and low birth weight infants (3,5). Although zinc deficiency disorder can have serious health implications among all age groups, infants are particularly vulnerable because their systemic zinc reserves are not fully developed and they are totally dependent on breast milk or formula. Therefore, the American Society for Clinical Nutrition recommends adding injectable zinc to PN for all infants and children, with priority for those who are premature, have low birth weight, or have chronic gastrointestinal dysfunction (4).

Zinc deficiencies among infants are difficult to identify for multiple reasons, including nonspecific signs and symptoms. The most common signs of zinc deficiency disorder include dermatitis and growth impairment, which can be attributed to multiple causes. Zinc deficiency disorder–associated dermatitis, which is a physical manifestation, is present in only the most severe cases. For premature infants, withdrawl of the amount of blood required to measure the serum zinc level might compromise the health of the infant; therefore, routine testing is not performed, which might explain, in part, why no other cases were reported.

Physicians who prescribe PN should recognize the potential risks for micronutrient deficiency, including zinc deficiency, among premature infants who require increased amounts or are unable to receive adequate doses. During shortages, clinicians might need to reserve micronutrients for the most vulnerable populations. According to FDA, shortages also are ongoing for other PN micronutrient components (e.g., selenium, chromium, and copper); FDA is working with manufacturers to prioritize which micronutrients to produce and to identify other sources for the micronutrients. Until the manufacture of these micronutrients increases, shortages will continue. Hospitals with limited stocks of injectable zinc should consider reserving available supplies for infants with the highest risk for deficiency. Whenever PN without the standard micronutrients is administered to patients, either as a result of shortages or other considerations, monitoring for signs and symptoms of micronutrient deficiencies is recommended. Health-care providers should always consider the specific clinical situation when applying these guidelines for individual clinical care.

## Figures and Tables

**TABLE:** Seven infants with physician-diagnosed zinc deficiency disorder, by selected characteristics — Washington, DC, and Houston, Texas, 2012–2013

Patient	Sex	Gestational age (wks)	Birth weight (g)	Cholestasis	Race/Ethnicity	Received PN	Dermatitis	PN duration before diagnosis (wks)	Serum zinc level (*μ*g/dL)*	Serum alkaline phosphatase level (U/L)†	Bacterial infection	Infant death
1	M	24	734	Yes	Black, non-Hispanic	Yes	Yes	10	NA	192	No	No
2	M	24	673	Yes	Black, non-Hispanic	Yes	Yes	15	14	62	No	No
3	F	29	551	Yes	Black, non-Hispanic	Yes	Yes	10	56	32	No	No
4	M	37	2,950	Yes	White, Hispanic	Yes	Yes	18	16	74	No	No
5	M	25	690	Yes	White, non-Hispanic	Yes	Yes	34	41	73	Yes§	Yes
6	F	37	2,620	Yes	White, Hispanic	Yes	Yes	5	25	110	Yes¶	Yes
7	M	33	1,599	Yes	Black, Hispanic	Yes	No	4	34	92	Yes**	No

**Abbreviations:** PN = parenteral nutrition; NA = not available.

* Lowest serum zinc level measured by laboratory testing; normal range = 70–120 *μ*g/dL.

† Lowest serum alkaline phosphatase level measured by laboratory testing; normal range = 150–420 U/L.

§ *Staphylococcus aureus*.

¶ Methicillin-resistant *Staphylococcus aureus.*

** *Escherichia coli* and *Kluyvera ascorbata.*
